# *Rankl* genetic deficiency and functional blockade undermine skeletal stem and progenitor cell differentiation

**DOI:** 10.1186/s13287-024-03803-3

**Published:** 2024-07-06

**Authors:** M. L. Schiavone, L. Crisafulli, C. Camisaschi, G. De Simone, F. R. Liberati, E. Palagano, N. Rucci, F. Ficara, Cristina Sobacchi

**Affiliations:** 1 IRCCS Humanitas Research Hospital, via Manzoni 56, Rozzano, Milan, 20089 Italy; 2 Institute for Genetic and Biomedical Research, Milan Unit, CNR, via Fantoli 16/15, Milan, 20138 Italy; 3 Flow Cytometry Core, IRCCS Humanitas Research Hospital, via Manzoni 56, Rozzano, Milan, 20089 Italy; 4 Institute of Biosciences and Bioresources, CNR, via Madonna Del Piano 10, Sesto Fiorentino, 50019 FI Italy; 5 Department of Biotechnological and Applied Clinical Sciences, University of L’Aquila, Via Vetoio - Coppito 2, L’Aquila, 67100 Italy

**Keywords:** RANKL, Skeletal stem cells, Differentiation, Osteopetrosis, Denosumab, Therapy

## Abstract

**Background:**

Skeletal Stem Cells (SSCs) are required for skeletal development, homeostasis, and repair. The perspective of their wide application in regenerative medicine approaches has supported research in this field, even though so far results in the clinic have not reached expectations, possibly due also to partial knowledge of intrinsic, potentially actionable SSC regulatory factors. Among them, the pleiotropic cytokine RANKL, with essential roles also in bone biology, is a candidate deserving deep investigation.

**Methods:**

To dissect the role of the RANKL cytokine in SSC biology, we performed ex vivo characterization of SSCs and downstream progenitors (SSPCs) in mice lacking Rankl (*Rankl*^*−/−*^) by means of cytofluorimetric sorting and analysis of SSC populations from different skeletal compartments, gene expression analysis, and in vitro osteogenic differentiation. In addition, we assessed the effect of the pharmacological treatment with the anti-RANKL blocking antibody Denosumab (approved for therapy in patients with pathological bone loss) on the osteogenic potential of bone marrow-derived stromal cells from human healthy subjects (hBMSCs).

**Results:**

We found that, regardless of the ossification type of bone, osteochondral SSCs had a higher frequency and impaired differentiation along the osteochondrogenic lineage in *Rankl*^*−/−*^ mice as compared to wild-type. *Rankl*^*−/−*^ mice also had increased frequency of committed osteochondrogenic and adipogenic progenitor cells deriving from perivascular SSCs. These changes were not due to the peculiar bone phenotype of increased density caused by lack of osteoclast resorption (defined osteopetrosis); indeed, they were not found in another osteopetrotic mouse model, i.e., the *oc/oc* mouse, and were therefore not due to osteopetrosis per se. In addition, *Rankl*^*−/−*^ SSCs and primary osteoblasts showed reduced mineralization capacity. Of note, hBMSCs treated in vitro with Denosumab had reduced osteogenic capacity compared to control cultures.

**Conclusions:**

We provide for the first time the characterization of SSPCs from mouse models of severe recessive osteopetrosis. We demonstrate that Rankl genetic deficiency in murine SSCs and functional blockade in hBMSCs reduce their osteogenic potential. Therefore, we propose that RANKL is an important regulatory factor of SSC features with translational relevance.

**Supplementary Information:**

The online version contains supplementary material available at 10.1186/s13287-024-03803-3.

## Background

Skeletal stem cells (SSCs) are a bone-specific subtype of somatic stem cells crucial for bone physiology [1]. They are endowed with self-renewal and multipotency capacity, and give rise to osteoblasts, chondrocytes, marrow stromal cells, and adipocytes in different proportions, depending on the compartment of origin (i.e., bone marrow, periosteum, growth plate) [2–6]. Gene expression analysis of SSCs and downstream progenitor populations identified ligands and receptors of signaling pathways that may modulate the activity of the SSCs and their progeny, and suggested a control based also on paracrine and/or autocrine molecular cues [7, 8]. Intrinsic regulatory factors may represent a means to tune the regenerative potential of mesenchymal progenitors, which is a highly pursued (but poorly achieved) goal due to the expected broad application in the field of regenerative medicine [9].

The Receptor Activator of NF-kB Ligand (RANKL) cytokine is recognized as a pleiotropic factor since its discovery; in fact, it was originally cloned by three independent groups and classified as a dendritic cell survival factor [10], a regulator of T cell function [11] and an essential osteoclast differentiation factor [12]. In line with this key function in the bone microenvironment, *Rankl* deficient (*Rankl*^*−/−*^) mice as well as patients bearing mutations in the *TNFSF11* gene (encoding RANKL) display severe osteopetrosis owing to lack of osteoclast formation [13, 14]. Several additional roles have been recognized for RANKL in pathophysiological conditions, making this cytokine an interesting target for therapy [15–19]. Of note, we previously reported that murine bone marrow-derived mesenchymal stromal cells (BMSCs) lacking Rankl (*Rankl*^*−/−*^) displayed a partial osteogenic defect that was corrected by restoring Rankl production through a lentiviral vector expressing the human soluble cytokine, thus pointing to a Rankl-mediated autocrine-paracrine loop in BMSCs [20]. In the context of that former paper, *Rankl*^*−/−*^ BMSCs were isolated by plastic adherence to cell culture plates, which likely resulted in a heterogeneous cell population. Driven by our earlier discovery, in the present study we sought to dig more deeply into the origin of the defect observed in *Rankl*^*−/−*^ BMSCs by analyzing a more homogeneous population. To this end, we took advantage of cytofluorimetric-based isolation protocols that allow the enrichment of skeletal stem and progenitor cell (SSPC) populations through specific surface markers. We applied these protocols in the *Rankl*^*−/−*^ mouse and in another model of severe recessive osteopetrosis, the *oc/oc* mouse, thereby providing for the first time a characterization of SSPCs in this pathological context. Phenotypic and functional evaluations established a clinically relevant crucial role for the RANKL cytokine in SSPC maintenance and differentiation. This conclusion was strengthened by the results of in vitro treatment of human BMSCs with the anti-RANKL blocking antibody Denosumab, which is a drug used in patients with pathological bone loss.

## Methods

### Animal models

*Rankl*^*+/−*^ mice were a kind gift of Prof. Yongwon Choi (University of Pennsylvania, Philadelphia, PA) [13]. Oc/+ mice fully backcrossed on the C57BL/6J background had been previously generated from the B6C3Fe a/a Tcirg1 oc/J-Ly5.2 mice originally purchased from Jackson Laboratory (Bar Harbor, ME) [21].

Both mouse colonies were maintained in heterozygosis in the specific pathogen-free facility of Humanitas Research Hospital; the litters were genotyped as described [13, 22]. *Rankl*^*−/−*^ and *oc/oc* mice and their respective wild-type (WT) littermates were euthanized by CO_2_ asphyxiation at the different post-natal ages indicated in the text; both males and females were included in the experimentation.

*Rankl*^*−/−*^ are indicated as knock-out (KO) in the Figures, for the sake of brevity.

Animal care and experimental procedures were performed in accordance with ethical rules of the Institutional Animal Care and Use Committee of Humanitas Research Hospital and with international laws (authorization n.11/2019-PR).

### Osteochondral skeletal stem and progenitor cell (ocSSPC) isolation and analysis

At necropsy, long bones (femurs and tibiae), hips and rib cage of *Rankl*^*−/−*^, oc/oc and age-matched C57BL6/J WT mice were cleaned from soft tissues, manually crushed, then subjected to enzymatic digestion in Hank’s Balanced Salt solution (HBSS) containing 1 mg/ml collagenase type IV, 4 mg/ml dispase, DNase 5 mg/ml (all from Sigma-Aldrich) and 0.025% trypsin (EuroClone SpA) for 30 min at 37 °C; the procedure was repeated 4 times. The digestion was then blocked using Fetal Bovine Serum (FBS; Lonza) and 0.5 M EDTA. The cells from all digests were pooled and stained with monoclonal anti-mouse CD45, Ter119, Tie2, AlphaV integrin (CD51), CD105, Thy1 (CD90), 6C3/BP-1 and CD200 fluorescent-conjugated antibodies, for fractionation by fluorescence activated-cell sorting (all the antibodies used in this staining are listed in Table S1). OcSSCs and downstream progenitors were defined as described by Chan and colleagues [7]. OcSSCs were sorted using a FACS Aria II cell sorter or analyzed using an LSR Fortessa flow cytometer (BD Biosciences) equipped with BD FACSDIVA™ software (BD Biosciences) and analyzed with FlowJo software v10.9 (FlowJo LLC, BD Biosciences).

In dedicated experiments, cells were sorted in PureZOL™ Reagent (Bio-Rad) for RNA isolation or in αMEM (Sigma-Aldrich) for subsequent culture.

The same procedure was applied for the analysis of ocSSPCs in the skull bone.

### Perivascular skeletal stem and progenitor cell (pvSSPC) isolation and analysis

The cell suspension for pvSSPC analysis was prepared as above described. After digestion the cells were stained using fluorochrome-conjugated antibodies against mouse CD45, CD31, CD140a (PDGFRa), Sca1, and CD24 for fractionation by FACS (all the antibodies used in this staining are listed in Table S2). PvSSCs and downstream progenitors were defined as described by Ambrosi and colleagues [8]. PvSSCs were isolated and analyzed with the same instruments and tools above described.

### Mineralization assay in clonogenic culture

Sorted ocSSCs were plated at clonal density in 6-well-plate, cultured for 7 days in α-MEM supplemented with 20% FBS and 1% Penicillin/Streptomycin (P/S), then put in osteogenic induction medium (OIM) made of α-MEM, 15% FBS, 50 µM ascorbic acid, 10 mM β-glycerophosphate, and 100 nM dexamethasone, and 1% P/S for 21 days. Finally, mineralizing clones were fixed with 70% EtOH for 1 h at RT, and stained with Alizarin Red (ARS) for 30 min at RT. The stain was extracted with acetic acid 10% and absorbance was measured at 405 nm on a Promega™ GloMax® Plate Reader.

### Primary calvarial osteoblast culture

The calvarial bone was dissected from WT and *Rankl*^*−/−*^ newborn mice (postnatal day 3–4), cleaned from adherent soft tissue, and sequentially digested in 1x HBSS (EuroClone SpA) containing 1 mg/ml collagenase type IV, 0.025% trypsin and 1% P/S at 37 °C. The cells from digests 2–4 were collected, pooled, and washed, then plated in α-MEM supplemented with 15% FBS and 1% P/S.

For the evaluation of the mineralization capacity, cells were plated in 24-well plates (4 × 10^4^ cells/well); OIM was added when cultures reached 80% confluence. After 7 and 14 days of osteogenic induction, cells were fixed and stained as above.

### Gene expression analysis

Total RNA was extracted from sorted ocSSCs as well as from primary osteoblasts cultured in basal condition and after 7 and 14 days of osteogenic induction, using the PureZOL™ Reagent (Bio-Rad) according to the manufacturer’s instructions. Reverse transcription was carried out using 1.0 µg total RNA and the High-Capacity cDNA Reverse Transcription Kit (Applied Biosystems). Quantitative PCR (qPCR) was performed using the SsoAdvanced™ SYBR® Green Supermix (Bio-Rad) and gene-specific primers (Table S3). The amplification was performed using the ViiA7 Real-Time PCR Detection System (Applied Biosystems) with the following cycling conditions: cDNA denaturation and polymerase activation at 95 °C for 20 seconds (s); 40 cycles of denaturation at 95 °C for 10 s and annealing at 60 °C for 20 s; extension for 60 cycles at 65 °C for 30 s and melting curve analysis step at 65 °C to 95 °C with 0.5 °C increment of 2 s/step. The relative gene expression analysis of target genes was conducted following the comparative 2^–ΔCt^ method and the normalized expression was calculated as arbitrary units (AU).

### Human bone marrow stromal cells (hBMSCs)

Human BMSCs from young healthy individuals of both sexes were purchased from Stem Cell Technologies (cat. #70,071).

Denosumab (Xgeva 150 mg, injectable, Amgen) was purchased through IRCCS Humanitas Research Hospital pharmacy. To assess Denosumab effect on BMSC viability, 20,000 cells were plated in 96-well-plate in DMEM low glucose medium and treated with 5 µg/ml Denosumab or isotype control (human IgG2, R&D) every 48 h. Viability was evaluated with the MTT assay (VWR international srl), according to the manufacturer’s instructions.

To assess Denosumab effect on BMSC osteogenic potential, 40,000 cells were plated in 48-well-plate in OIM added with 5 µg/ml Denosumab or isotype control; culture medium was changed twice a week. After two weeks, ARS was performed according to standard protocols. In dedicated wells, RNA isolation and gene expression analysis were carried out as above described; the gene-specific primers used are listed in Table S4.

### Statistical analysis

Statistical analysis was carried out using GraphPad Prism version 9.0 (GraphPad Software). Data were expressed as mean ± standard error of the mean (SEM). Mann-Whitney test for single comparison or Kruskall-Wallis and Friedmann for multiple comparison were used to calculate *p*-values. Significance is represented as follows: * *p* < 0.05, ** *p* < 0.01, *** *p* < 0.001.

## Results

### Dissection of SSPC populations unveils alterations in their hierarchy in *Rankl*^*−/−*^ mice

**Fig. 1:**
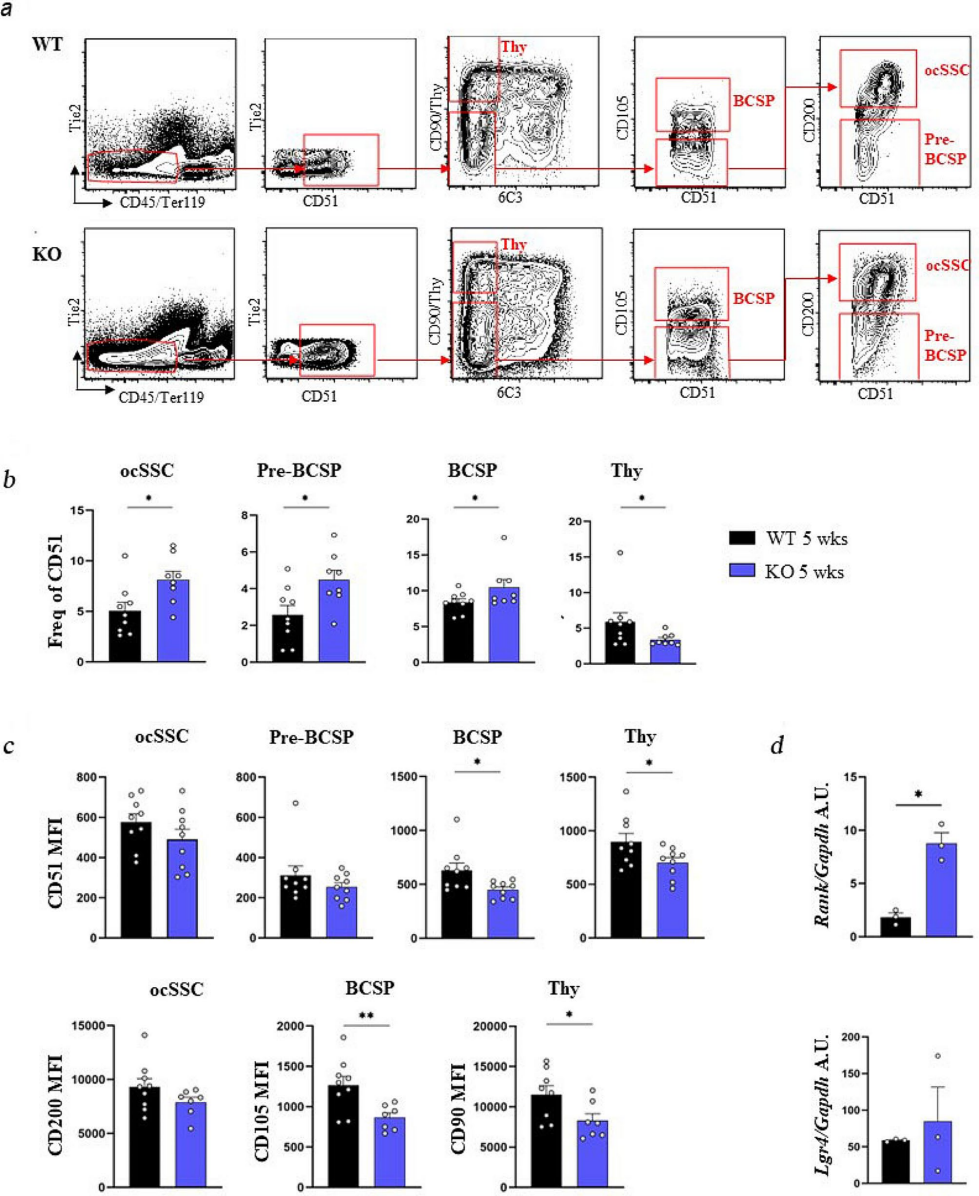
FACS sorting and characterization of osteochondral SSC (ocSSCs) and downstream progenitors in WT and *Rankl*^*−/−*^ mice. **a** Representative contour plots of the gating strategy for both genotypes. **b** Abundance of the different ocSSPC populations, expressed as frequency of CD51^+^ cells, in 5-week-old mice of both genotypes. **c** Expression level of the positive markers in the indicated cell populations, expressed as mean fluorescence intensity (MFI). **d** Gene expression level of the RANKL receptors *Rank* and *Lgr4* in FACS-sorted ocSSCs, normalized on *Gapdh* and expressed as Arbitrary Units (A.U.). All the data are expressed as mean ± SEM. * *p* < 0.05, ** *p* < 0.01; Mann-Whitney test. KO: knockout, *Rankl*^*−/−*^

To assess whether *Rankl* expression is relevant to SSC hierarchy, we assessed in vivo the frequency of SSCs and downstream progenitors in mice with *Rankl* constitutive genetic deficiency [13]. In detail, we produced cell suspensions by mechanical dissociation and enzymatic digestion of bones (including hindlimbs, ribs and sternum) of 5-week-old *Rankl*^*−/−*^ and WT mice. We obtained 20 ± 10 millions and 50 ± 10 millions of total cells from *Rankl*^*−/−*^ and WT mice, respectively, in line with the mean 2.5-fold difference in body weight for the two genetic backgrounds. The cells were stained with a mix of specific antibodies for FACS analysis previously reported to allow the isolation of highly pure, postnatal SSCs and downstream progenitors of bone, cartilage, and stromal tissue [7]. The abundance of the different subsets of skeletal progenitors was expressed as frequency of CD51^+^ cells, since CD51 is the earlier positive marker among those used for the dissection of the SSC hierarchy and is common to all the subpopulations (Fig. 1a); importantly, the percentage of CD51^+^ cells within the CD45^−^Ter119^−^Tie2^−^ non-hematopoietic cells was the same in *Rankl*^*−/−*^ and WT mice. FACS analysis revealed that the more immature population (osteochondral SSCs, ocSSCs, identified as CD45^−^Ter119^−^Tie2^−^CD51^+^Thy^−^6C3^−^CD105^−^CD200^+^) as well as the downstream pre-bone, cartilage and stromal progenitors (preBCSPs, identified as CD45^−^Ter119^−^Tie2^−^CD51^+^Thy^−^6C3^−^CD105^−^CD200^−^) and BCSPs (CD45^−^Ter119^−^Tie2^−^CD51^+^Thy^−^6C3^−^CD105^+^) were significantly increased in *Rankl*^*−/−*^ compared to WT mice, while the opposite was observed for the Thy subpopulation (CD45^−^Ter119^−^Tie2^−^CD51^+^Thy^+^6C3^−^CD105^+^), which was more abundant in the WT (Fig. 1b).

In parallel, we found that CD51 expression was lower in *Rankl*^*−/−*^ BCSPs and Thy cells compared to the corresponding subpopulations in the WT, and similarly CD105 and Thy/CD90 expression was lower in *Rankl*^*−/−*^ BCSPs and Thy cells, respectively, than in the same subpopulations in the WT (Fig. 1c).

In our previous work, we proposed that the absence of Rankl resulted in the impairment of an autocrine/paracrine signaling in BMSCs [20]. In line with this hypothesis, we found that the isolated SSCs expressed both Rankl receptors *Rank* and *Lgr4*, and that the former was significantly higher in *Rankl*^*−/−*^ SSCs compared to WT (Fig. 1d), possibly as an attempt to compensate defective signaling due to absence of the ligand.

### The altered frequency of skeletal stem and progenitor cells (SSPCs) in *Rankl*^*−/−*^ mice does derive from the absence of RANKL

The *Rankl*^*−/−*^ mouse is a well-known model of autosomal recessive osteopetrosis, a rare skeletal genetic disease characterized by extremely dense bone and reduced marrow space. The skeletal defects worsen with age [23, 24]; therefore, to establish whether the phenotype observed in the SSPCs was a distinctive characteristic of *Rankl*^*−/−*^ SSPCs or was secondary to progressive alteration of the bone microenvironment, we analyzed the SSC hierarchy in WT and *Rankl*^*−/−*^ mice at younger age, when the bone pathology is less severe.

**Fig. 2:**
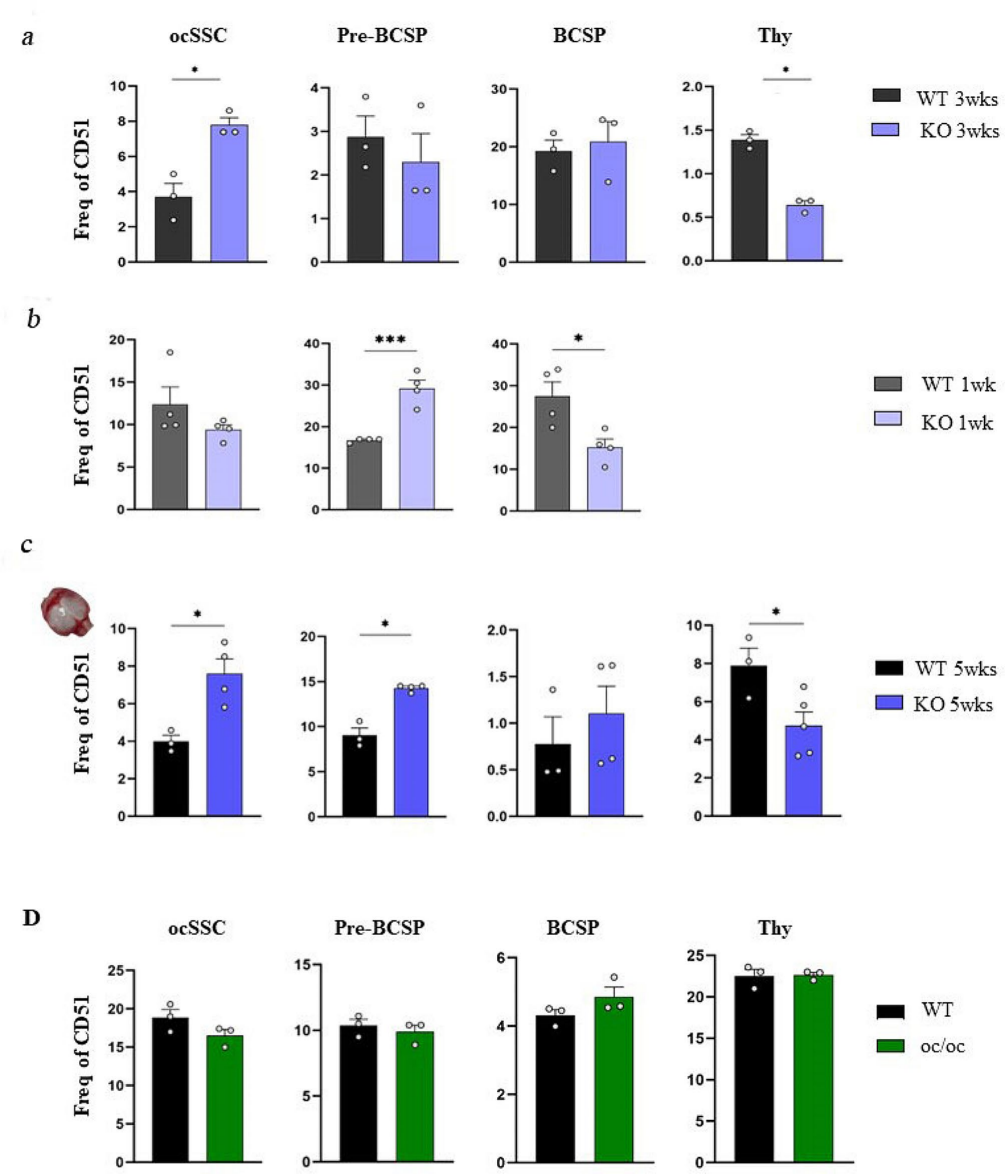
Comparative analysis of ocSSCs and downstream progenitors in WT and *Rankl*^*−/−*^ mice at different ages and in different skeletal compartments, and in *oc/oc* mice. **a** Abundance of the different ocSSPC populations, expressed as frequency of CD51^+^ cells, in 3-week-old WT and *Rankl*^*−/−*^ mice. **b** Analysis of ocSSPCs in 1-week-old WT and *Rankl*^*−/−*^ mice. **c** Analysis of ocSSPCs isolated from the skull of 5-week-old WT and *Rankl*^*−/−*^ mice. **d** Abundance of the different ocSSPC populations in WT and *oc/oc* mice. All the data are expressed as mean ± SEM. * *p* < 0.05, *** *p* < 0.001; Mann-Whitney test. KO: knockout, *Rankl*^*−/−*^

At 3 weeks of age, tissue processing following the protocol above described yielded 15 ± 5 millions and 20 ± 5 millions of total cells in *Rankl*^*−/−*^ and WT mice, respectively; also at this age, the ratio of total cell number was in line with the 1.5-fold difference in body weight between the two genotypes. FACS analysis highlighted again higher SSC and lower Thy^+^ subpopulation frequency in *Rankl*^*−/−*^ mice compared to WT (Fig. 2a), while no difference was observed in the intermediate subpopulations. At a younger age (1 week), the same number of cells were collected from mice of the two genotypes (10 million), which in fact had similar body size and weight. Of note, the frequency of SSCs did not differ between *Rankl*^*−/−*^ and WT mice at 1 week of age, while preBCSPs were significantly increased and BCSPs significantly decreased in the knock-out compared to controls (Fig. 2b). We could not reliably detect the Thy^+^ subpopulation at this stage owing to the very low number of cells in this gate. These results demonstrated that altered proportions in the SSC hierarchy were present since early life in *Rankl*^*−/−*^ mice, thus arguably inherent to a differentiation defect of SSCs in the framework of *Rankl* deficiency.

To assess whether these observations were related to the developmental origin of the bones analyzed, we characterized SSPCs in the skull of *Rankl*^*−/−*^ and WT mice. Indeed, the skull bones originate through intramembranous ossification, at variance with the long bones which derive from endochondral ossification. Importantly, the presence in the skull of cells with the immunophenotype adopted here to define SSPCs has been recently demonstrated [4]. Based on this, we processed the skull of 5-week-old *Rankl*^*−/−*^ and WT mice with the same protocol as described above for long bones. Again, we found that SSCs and preBCSPs were more and Thy^+^ less abundant in *Rankl*^*−/−*^ than in WT mice (Fig. 2c), and that preBCSP and Thy^+^ had a lower expression of the CD51 marker as compared to WT (Fig. S1); on the contrary, CD200, CD105 and CD90 surface markers had similar expression level in WT and *Rankl*^*−/−*^ SSPCs. These data indicated that changes in the frequency of the different SSPC populations were not biased by the developmental origin of bones.

In *Rankl*^*−/−*^ osteopetrosis the skeletal pathology is caused by absent osteoclast formation owing to lack of RANKL [25]. Both in mice and in patients several osteopetrosis subtypes exist, with different etiopathogenetic mechanisms based on the specific affected gene [26]. So, we asked whether the altered frequency of SSPCs found in *Rankl*^*−/−*^ mice was due to the osteopetrotic environment per se, and thus might be a feature present also in other murine models of the disease. To answer this question, we analyzed SSCs and downstream progenitors from *oc/oc* mice, as the animal model of the most frequent subset of human recessive osteoclast-rich osteopetrosis associated with mutations in the *TCIRG1* gene [27]. After bone digestion, we obtained 10 ± 3 millions and 5 ± 2 millions of total cells from WT and *oc/oc* mice, respectively, and the ratio between the two groups in terms of cellular yield was the same as their ratio in body weight. Interestingly, we did not find any change in the frequency of the diverse cell populations in *oc/oc* mice as compared to age-matched (i.e., 2-week-old) WT controls (Fig. 2d). This demonstrated that the defect observed in the *Rankl*^*−/−*^ mouse was specifically associated with the lack of the RANKL cytokine.

### *Rankl* genetic deficiency also impairs the perivascular SSC progeny in *Rankl*^*−/−*^ mice

**Fig. 3:**
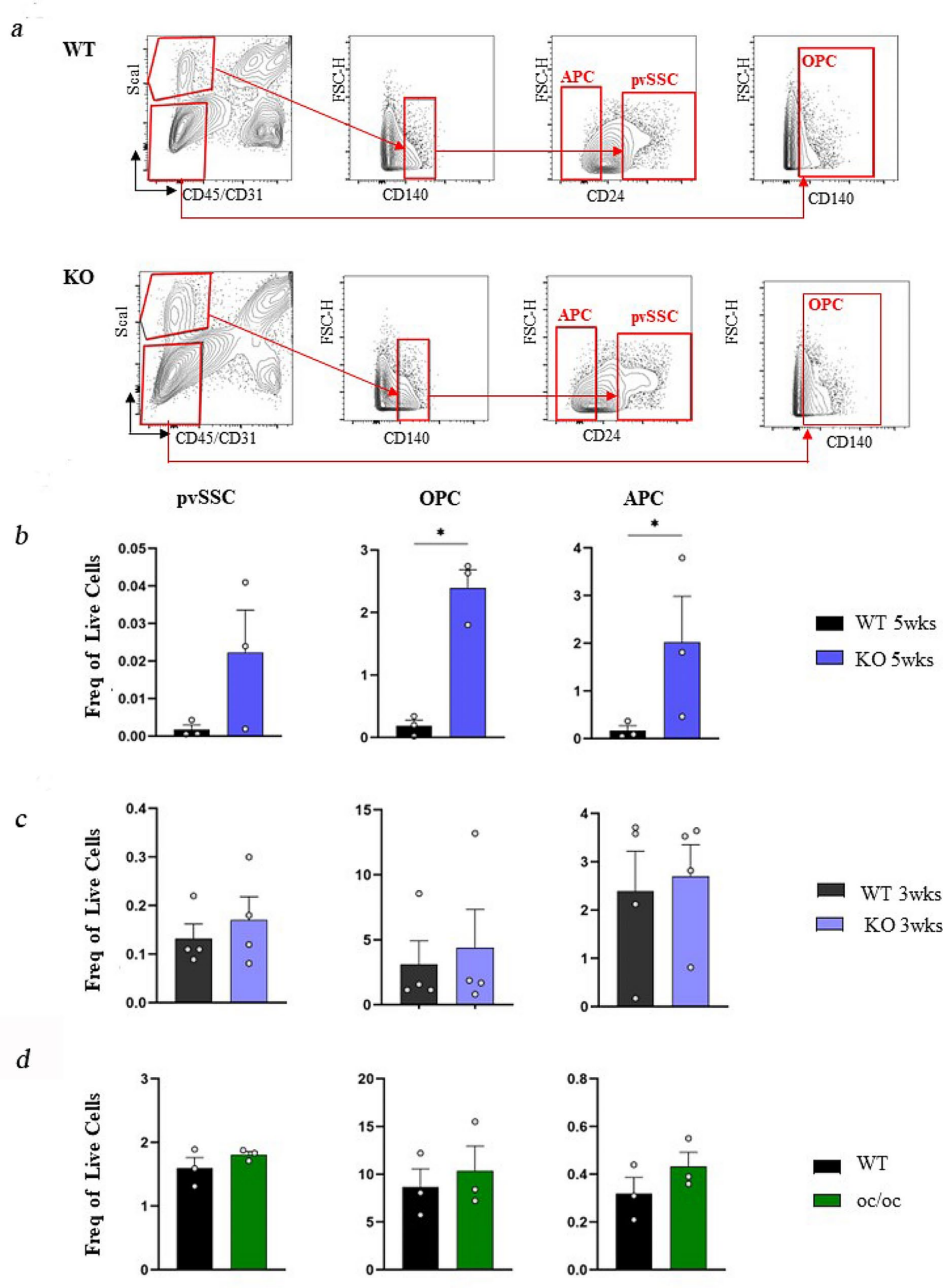
FACS sorting and characterization of perivascular SSC (pvSSC) and downstream progenitors. **a** Representative contour plots of the gating strategy for WT and *Rankl*^*−/−*^ mice. **b** Abundance of the different pvSSPC populations, expressed as frequency of live cells, in 5-week-old WT and *Rankl*^*−/−*^ mice. **c** Analysis of pvSSPCs in 3-week-old WT and *Rankl*^*−/−*^ mice. **d** Abundance of the different pvSSPC populations in WT and *oc/oc* mice. All the data are expressed as mean ± SEM. * *p* < 0.05; Mann-Whitney test. KO: knockout, *Rankl*^*−/−*^

SSCs are indeed a more pure and defined cell population as compared to BMSCs; still, specific SSC populations can be distinguished, particularly based on their location [28]. Perivascular (pv)SSCs have recently been described as an SSC subtype distinct from the ocSSCs described above. Specifically, pvSSCs arise later during skeletal development and display a different localization, a separate gene expression profile and panel of surface markers, a pronounced adipogenic potential and capacity to support hematopoiesis [8]. Therefore, we wondered whether lack of RANKL also affected this cell population. To answer this question, we sorted and analyzed pvSSCs in 5-week-old WT and *Rankl*^*−/−*^ mice. In detail, we stained the cell suspension deriving from the same digestion procedure described for the ocSSCs, with a different mix of antibodies that allows identifying pvSSCs as CD45^−^CD31^−^CD140a^+^Sca1^+^CD24^+^ cells; osteochondrogenic progenitor cells (OPCs) as CD45^−^CD31^−^CD140a^+^Sca1^−^cells; and adipogenic progenitor cells (APCs) as CD45^−^CD31^−^CD140a^+^Sca1^+^CD24^−^ [8] (Fig. 3a). In general, pvSSCs constituted a very rare cell population in both WT and *Rankl*^*−/−*^ mice at 5 weeks of age. The frequency of pvSSCs showed a trend to increase in *Rankl*^*−/−*^ compared to WT mice, while OPCs and APCs were frankly significantly higher in knock-out mice (Fig. 3b). Of note, the expansion of APCs could be interpreted in line with our previous observation of a trend towards increased in vitro adipogenic potential in *Rankl*^*−/−*^ BMSCs compared to WT [20]. On the other hand, no alteration of the pvSSC hierarchy was detected in *Rankl*^*−/−*^ mice at 3 weeks of age (Fig. 3c) or in *oc/oc* mice compared to age-matched controls (Fig. 3d).

Overall, these results strengthened the conclusion that the alterations found in SSPC populations in *Rankl*^*−/−*^ mice were specifically dependent on the absence of RANKL.

### *Rankl* deficiency impairs the osteogenic potential of ocSSCs and osteoblasts

**Fig. 4:**
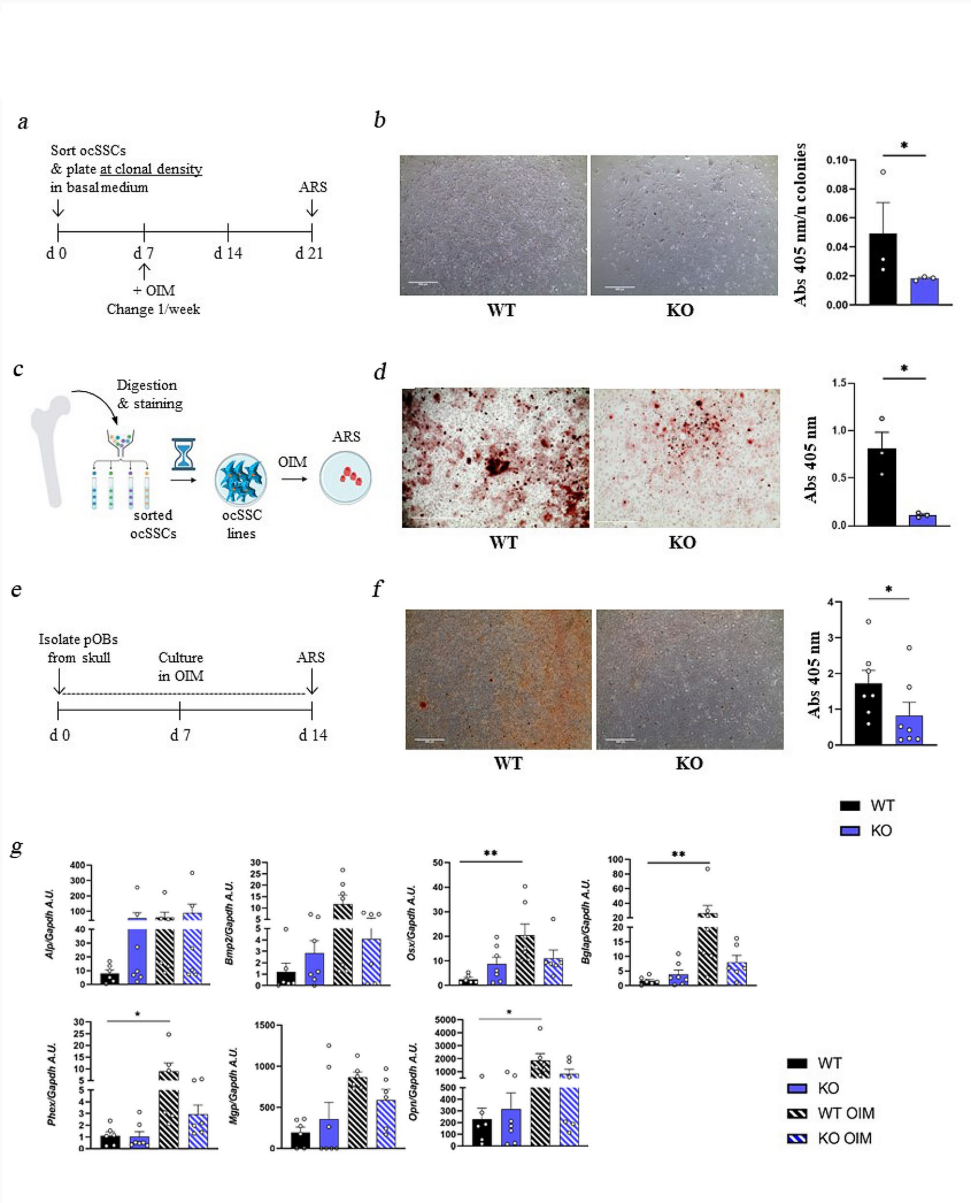
Characterization of the osteogenic potential of WT and *Rankl*^*−/−*^ ocSSCs and primary osteoblasts. **a** Schematic representation of the experimental protocol for the assessment of the osteogenic potential of ocSSCs in clonogenic conditions. Briefly, *Rankl*^*−/−*^ and WT ocSSCs were plated immediately after sorting at clonal density in basal medium. After 7 days, the cultures were exposed to OIM for 3 weeks. **b** Representative images of Alizarin Red Staining (ARS) at the end of osteogenic induction of the cultures described in **a**. The stain was chemically extracted, quantified by absorbance (Abs) reading at 405 nm and normalized on the number of colonies formed. **c** Schematic representation of the experimental protocol for the assessment of the osteogenic potential of WT and *Rankl*^*−/−*^ ocSSC lines. Briefly, after in vitro expansion of sorted *Rankl*^*−/−*^ and WT ocSSCs, the cell lines obtained were treated with OIM for 3 weeks. **d** Representative images of ARS at the end of osteogenic induction of the cultures described in **c** and quantization of the extracted stain by Abs reading at 405 nm. **e** Schematic representation of the experimental protocol for the assessment of the osteogenic potential of WT and *Rankl*^*−/−*^ osteoblasts. Briefly, *Rankl*^*−/−*^ and WT primary osteoblast cultures were established and induced to mineralize according to standard protocols. **f** Representative images of ARS at the end of osteogenic induction of the cultures described in **e** and quantization as in **d**. Scale bar in b, d and f: 500 μm. Higher magnifications of this panels are provided in Figure S2. **g** Gene expression analysis of representative osteogenic genes in WT and *Rankl*^*−/−*^ pre- and mature osteoblasts, normalized on *Gapdh* and expressed as Arbitrary Units (A.U.). All the data are expressed as mean ± SEM. * *p* < 0.05; b, d, f: Mann-Whitney test; g: Kruskal-Wallis test. OIM: Osteogenic Induction Medium. pOBs: primary osteoblasts. KO: knockout, *Rankl*^*−/−*^

To assess whether the reduced frequency of the Thy^+^ population in *Rankl*^*−/−*^ mice was accompanied by a functional defect, we tested the mineralization capacity of *Rankl*^*−/−*^ vs. WT ocSSCs at *bona fide* single cell level. In detail, immediately after sorting, *Rankl*^*−/−*^ and WT ocSSCs were plated at clonal density and cultured for 7 days in basal medium, then shifted in osteogenic induction medium (OIM) for 3 weeks. At the end of the culture, mineralization was evaluated by Alizarin Red Staining (ARS) and quantization, according to standard procedures (Fig. 4a); values were normalized to the number of colonies obtained. This experimental setting showed a significantly reduced osteogenic capacity in *Rankl*^*−/−*^ compared to WT ocSSCs (Fig. 4b and Fig. S2a). We then repeated the experiment using SSC lines established in culture after sorting (Fig. 4c). In this case, cells were exposed to osteogenic induction medium following standard protocols [20]. Also in this condition, we observed significantly reduced mineral deposition (Fig. 4d and Fig. S2b). Finally, we performed the mineralization assay using primary osteoblasts isolated from single calvariae of 3-day-old *Rankl*^*−/−*^ and WT mice (Fig. 4e). These cells are generally regarded as pre-osteoblasts, i.e., committed cells falling in between osteoprogenitors and mature osteoblasts, and their terminal differentiation is achieved by culture in OIM [29]. We observed a significant reduction in the mineralization capacity of *Rankl*^*−/−*^ vs. WT osteoblasts after 2 weeks of osteogenic induction (Fig. 4f and Fig. S2c), as well as at an earlier time point (i.e., 7 days; Fig. S3a), in line with our previous report in *Rankl*^*−/−*^ BMSCs compared to WT [20]. We did not find significant difference between WT and KO in the expression of the osteoblast marker genes investigated in basal condition and after 7 and 14 days of osteogenic induction; however, it was noticeable that in the KO they did not undergo a clear upregulation upon osteogenic induction (Fig. 4g and Fig. S3b). At both time points, Western blot analysis showed the expression of Lgr4 and Rank proteins in WT and *Rankl*^*−/−*^ osteoblasts (Fig. S3c), supporting the hypothesis of Rankl signaling through its receptors in cells of the osteogenic lineage.

Altogether, these results confirmed our previous observation of a significant reduction of the osteogenic potential of *Rankl*^*−/−*^ BMSCs compared to WT, in vivo and in vitro [20].

Of note, an earlier work had shown no change in extracellular matrix mineralization, alkaline phosphatase activity and osteocalcin production in primary osteoblasts isolated from *oc/oc* mice compared to WT, thus ruling out the presence of an intrinsic osteoblast defect in *oc/oc* mice [30]. This discrepancy between the *Rankl*^*−/−*^ and the *oc/oc* model further confirm that the cell phenotype in the former is specifically related to absence of RANKL and strengthen the hypothesis of a non-dispensable role of this cytokine in the osteogenic lineage.

**Fig. 5:**
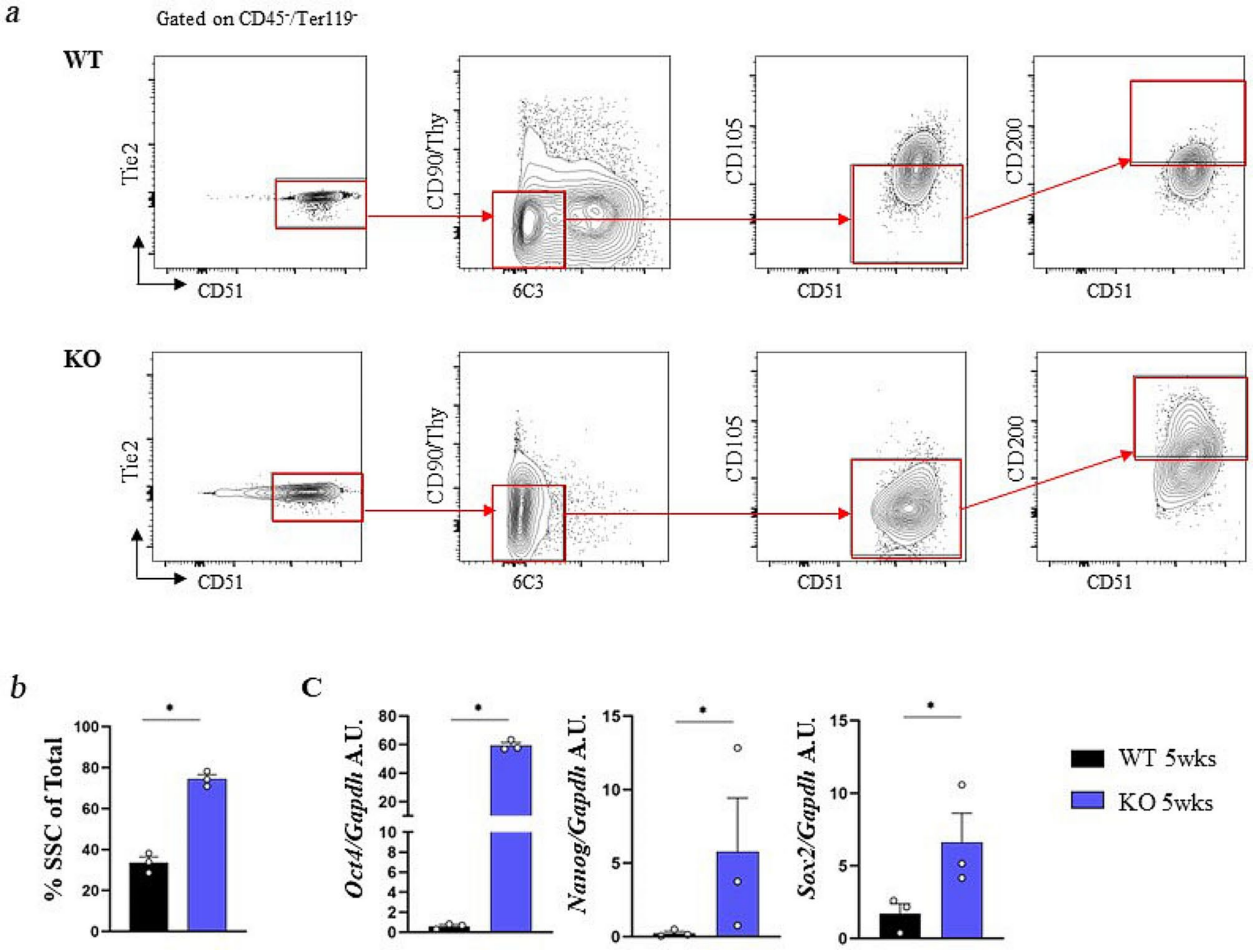
Expression profile of WT and *Rankl*^*−/−*^ ocSSC lines. **a** Representative contour plots of the gating strategy for FACS analysis of WT and *Rankl*^*−/−*^ ocSSC lines, using the same surface markers exploited for their isolation from bone cell suspensions. Cell populations were gated on CD45^−^/Ter119^−^; as expected, all cells were negative for these markers. **b** Frequency of cells displaying the immunophenotype of SSCs (as defined by Chan et al., 2015 and adopted in this work) after the establishment of WT and *Rankl*^*−/−*^ ocSSC lines. **c** Gene expression analysis of representative stemness markers in WT and *Rankl*^*−/−*^ ocSSC lines, normalized on *Gapdh* and expressed as Arbitrary Units (A.U.). All the data are expressed as mean ± SEM. * *p* < 0.05; Kruskal-Wallis test. KO: knockout, *Rankl*^*−/−*^

The reduced osteogenic potential in the *Rankl*^*−/−*^ mouse might result from the defective differentiation capacity of SSCs along the osteogenic lineage, leading to the expansion of the more immature populations upstream of the bottleneck at the transition from BCSPs to Thy^+^ cells (previously shown in Fig. 1b). To verify this hypothesis, we assessed the expression of the markers used for FACS sorting of the SSCs after long term culture. It is extensively documented that, despite optimized culture conditions, adult stem cells gradually lose their stem cell properties with in vitro passaging and tend to differentiate [31]. So, it was not surprising to see that in WT cultures, in basal condition (i.e., in the absence of osteogenic induction), a percentage of cells acquired the expression of CD105 and 6C3 markers (which were not expressed by the SSCs originally sorted), likely proceeding towards a more differentiation-prone status (Fig. 5a). Interestingly, the original immunophenotype was better preserved and the percentage of SSCs on total cells in culture higher in *Rankl*^*−/−*^ compared to WT cultures (Fig. 5a, b). Also, *Rankl*^*−/−*^ SSC lines showed higher expression of the stemness marker genes *Oct4*, *Nanog* and *Sox2* compared to WT ones (Fig. 5c). These results indicated that WT SSC cultures were more heterogeneous than *Rankl*^*−/−*^ ones and contained a fraction of less undifferentiated cells ready to promptly respond to osteogenic induction, which in fact resulted in greater mineralization upon stimulation (previously shown in Fig. 4). On the contrary, *Rankl*^*−/−*^ SSCs were restrained in their differentiation, and this was due to a cell-autonomous defect related to *Rankl* deficiency and not secondary to a pathological environment.

### Treatment of human BMSCs with the anti-RANKL antibody reduces their osteogenic capacity

RANKL is a target of therapy in patients with diseases characterized by excessive bone loss, such as osteoporosis. This occurs more frequently in the elderly, which is physiologically accompanied by altered stem cell behavior in the SSC (and hematopoietic stem cells, as well) compartment. Therefore, we sought to confirm RANKL involvement in these cellular mechanisms also by evaluating the impact of prolonged RANKL blockade in human BMSCs.

**Fig. 6:**
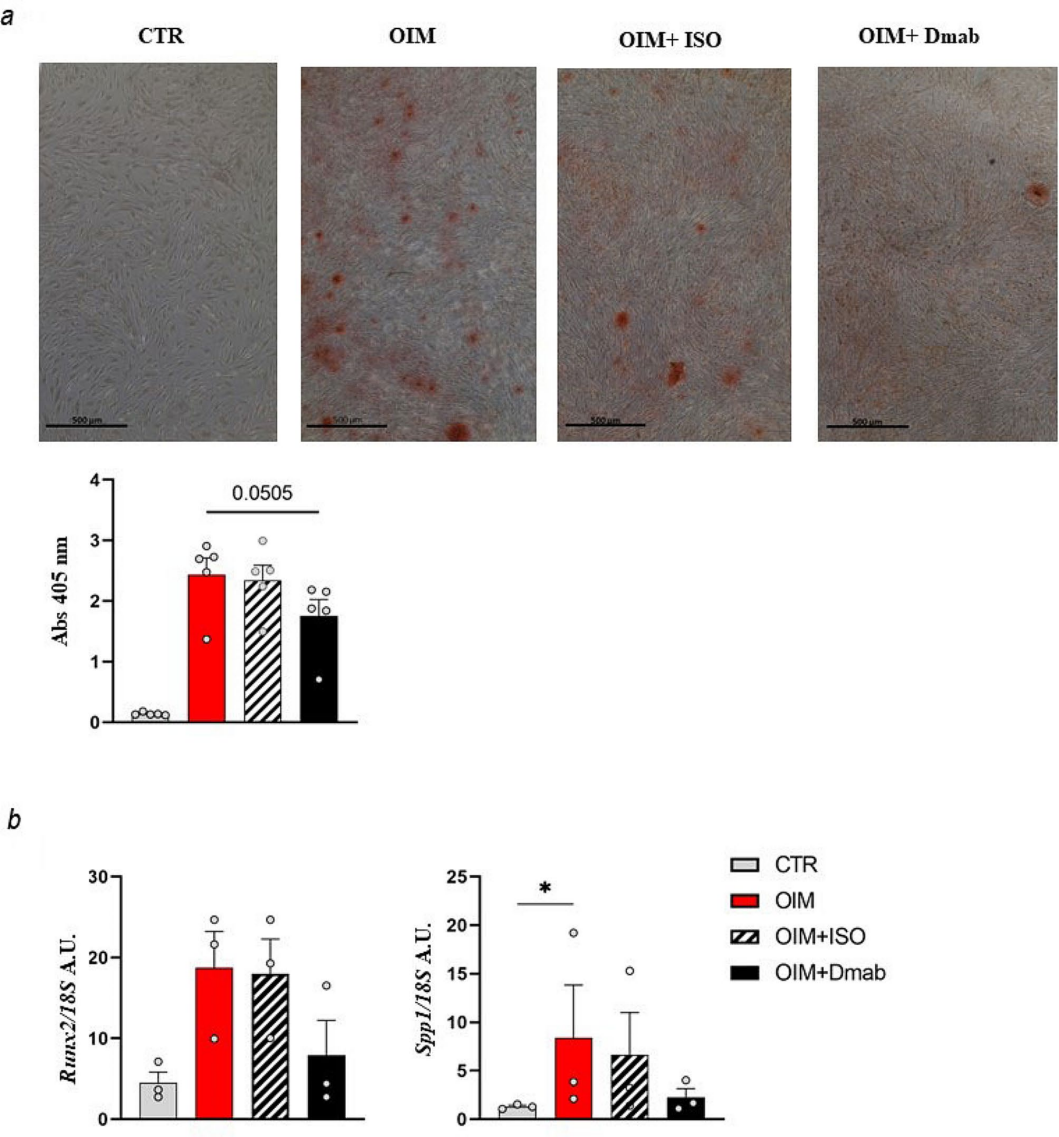
Impact of Denosumab treatment on the mineralization capacity of human BMSCs. **a** Representative image of ARS-stained cultures of human BMSCs from healthy donors after 2 weeks of culture either in basal medium (CTR) or in OIM alone or in OIM + isotype control (OIM + ISO) or in OIM + Denosumab (OIM + Dmab); in all the conditions, the culture medium was changed twice a week. At the end, the mineral deposition was quantified and indicated by Abs reading at 405 nm. Scale bar: 500 μm. Higher magnifications are provided in Figure S2. **b** Gene expression analysis of representative osteogenic genes in human BMSCs in the different treatment conditions indicated; normalization on *18 S.* Results are expressed as Arbitrary Units (A.U.). All the data are represented as mean ± SEM. * *p* < 0.05; a: Friedman test; b: Kruskal-Wallis test. CTR: control. OIM: Osteogenic Induction Medium. ISO: isotype control. Dmab: Denosumab

In detail, BMSCs from healthy donors were cultured in OIM in the presence of Denosumab (Dmab), the monoclonal antibody specifically targeting human RANKL used in the clinical setting, for 2 weeks; in concurrent osteogenic cultures, BMSCs were treated with the isotype antibody, as a control. We used a drug concentration (5 µg/ml twice a week) comparable to that administered in previous preclinical studies [32] and to the maximum plasmatic concentration (Cmax) value determined through pharmacokinetic studies in healthy individuals (i.e., 6.75 µg/ml when 60 mg of Dmab were subcutaneously administered to healthy subjects after fasting for 12 h [33]). Importantly, this treatment regimen did not affect BMSC viability, determined through MTT assay at different timepoints during osteogenic induction in the presence/absence of Dmab or in the presence of the isotype control (Fig. S4a). After 2 weeks of osteogenic induction in these conditions, mineralization was assessed with ARS staining and spectrophotometric evaluation, as above. We found that BMSCs treated with Dmab during osteogenic induction produced significantly less mineralized matrix than BMSCs in standard osteogenic conditions (Fig. 6a and Fig. S4b); on the contrary, the isotype antibody did not impact on the mineralization potential, indicating that the effect observed in the presence of Dmab was specifically due to RANKL blockade. Accordingly, the expression level of osteogenic genes was lower in BMSCs treated with Dmab (Fig. 6b). These results demonstrated that in this experimental setting RANKL blockade was detrimental to the osteogenic function of human BMSCs.

## Discussion

Skeletal stem and progenitor cell (SSPC) populations are crucial for bone physiology [1]. Possibly owing to the peculiarities of the bone microenvironment, their characterization has remained very limited for a long time [34]. Lineage tracing [2, 6, 35–37], single cell RNA sequencing [38–41] and FACS sorting of cells by means of extended panels of surface markers [7, 8, 42] have recently shed some light in the field. Nowadays, different SSC subtypes are recognized but molecular mechanisms are only partially dissected [43], consistently with the involvement of novel players [44].

In this framework, we propose that RANKL, known for its role as essential osteoclastogenic factor, besides those in immunity, mammary gland development and central thermoregulation (just to mention a few; [19]), is involved also in the maintenance of stemness features of SSCs, comprising self-renewal and multilineage differentiation capacity. This concept, initially essentially based on in vitro results using *Rankl*^*−/−*^ and WT BMSCs [20], is corroborated here by the ex vivo evidence on freshly sorted, pure SSPCs which provide a snapshot of cell populations occurring in vivo in the bone microenvironment. Indeed, here we demonstrated significant increase in the frequency of SSCs and reduction in the Thy^+^ population in *Rankl*^*−/−*^ mice compared to WT counterparts. The altered composition of the SSC lineage was documented in *Rankl*^*−/−*^ mice as early as in neonatal life (7 days after birth) and was not found in the *oc/oc* mouse model of osteopetrosis; thus, it was not a generic outcome of the osteopetrotic environment, rather an inherent characteristic of *Rankl*^*−/−*^ SSPCs. The expression of both RANKL receptors *Rank* and *Lgr4* by ocSSCs indicate that RANKL may modulate SSC properties acting directly through its receptor(s) in an autocrine-paracrine loop. In this scenario, upregulation of *Rank* in *Rankl*^*−/−*^ SSCs might be a compensatory mechanism due to lack of the ligand.

To dissect the complexity of SSC subsets in the framework of RANKL deficiency, we also characterized pvSSCs and their progeny, which comprises osteochondrogenic and adipogenic progenitors (OPC and APC, respectively). Both these populations were significantly increased in *Rankl*^*−/−*^ mice, and this was at variance with the reduction of the most differentiated cell population (Thy^+^) analyzed downstream ocSSCs. These opposite findings might be due to the different molecular programs activated in the ocSSC and pvSSC lineages [8], and indicate that Rankl role is context-dependent, in line with the concept of distinct niches and niche-specific factors in the bone microenvironment [45, 46]. The higher abundance of OPC in *Rankl*^*−/−*^ mice might be an attempt to compensate for the reduction of Thy^+^ progenitors along a distinct differentiation path. Indeed, not only is the differentiation potential of ocSSCs defective; their function is flawed too, and we demonstrated a reduced mineralization capacity in *Rankl*^*−/−*^ ocSSCs plated at clonal density or as a bulk population, after the establishment of SSC lines, as well as in *Rankl*^*−/−*^ primary osteoblasts. One might argue that this osteogenic defect is not apparent in vivo; in fact, *Rankl*^*−/−*^ mice have markedly increased, not reduced, bone mass. On the other hand, *Rankl*^*−/−*^ mice do not have osteoclasts in their bones (which in fact causes the osteopetrotic phenotype); it is reasonably expected that the complete absence of the unique cell type able to resorb bone largely prevails over a partial defect in the bone-forming function. Based on the gene expression data of *Rankl*^*−/−*^ versus WT osteoblasts, it may also be expected that not only the amount, but also the composition of the bone matrix produced by osteogenic cells lacking *Rankl* differs from the WT, resulting in lower bone quality.

Our results also show that Rankl deficiency impacts on the transcriptional profile of SSPCs, indeed we observed lower expression of CD51, CD105 and CD90/Thy in the subpopulations expressing these markers in *Rankl*^*−/−*^ mice. In diverse contexts, CD51 expression has been correlated with the acquisition/maintenance of stemness features [47, 48]. With respect to osteoprogenitor cells, CD51 expression has been associated with proliferative and migratory potential [49], and with regenerative capacity [50]. On our hand, we did not have evidence of differences in proliferation between *Rankl*^*−/−*^ and WT SSCs, while other functions still must be investigated.

CD105 and CD90 have been included in the panel of surface markers distinctive of human MSCs since early studies in the field [51]. Of note, a reduction in CD90 expression has been reported to result in increased differentiation of in vitro expanded human MSCs [52]. The different species and cell population analyzed may be responsible for the discrepancy between our results and those described by Lv and colleagues. Moreover, we may speculate that in *Rankl*^*−/−*^ ocSSCs the differentiation defect arises at an early stage in the ocSSC hierarchy. Indeed, CD51, which is upstream of CD90 in the panel of markers used for FACS sorting and population dissection, was reduced in all the subsets where it is expressed; this might determine a defect hard to overcome.

Overall, our study is relevant from diverse points of view. First, it investigated the role of RANKL with respect to SSC stemness features, which was unprecedented, to the best of our knowledge. Indeed, the *Rankl* gene has been inactivated at several levels through the Cre-loxP technology driven by diverse stage-specific promoters, including also the *Prrx1* gene promoter, in the framework of studies aimed at defining the contribution of different populations along the osteogenic lineage to the regulation of osteoclast formation through RANKL production, so studies having a different purpose as compared to our own [53]. Prx1 was originally demonstrated to regulate the development of specific skeletal elements (i.e., the limbs and parts of the skull) since very early stages in the embryo, so deemed to be an early mesenchymal lineage marker [54]. On the other hand, Prx1 was later found to be required in a different compartment, i.e., vascular development in the lung [55]. In addition, Ambrosi and colleagues showed that, in the *Prx1-*Cre: mTmG reporter mouse, the labeling of inguinal white adipose tissue and skeletal muscle-resident CD45^−^CD31^−^Sca1^+^ cells was comparable to that in bone, indicating that Prx1 was indeed an MSC marker, though likely not specific for SSCs [56]. Finally, Prx1 has recently been found to act as a master transcription factor in the myofibroblastic lineage progression of stromal fibroblasts [57]. Based on these data, the *Prrx1* promoter does not selectively mark SSCs, a function that in fact is accomplished by no single genetic lineage driver [43].

We sorted SSPCs in the constitutive *Rankl* KO mouse model available in our lab by means of established large panels of cell surface markers defining two specific subsets of stem cells [7, 8]. In this way, we unveiled the role of RANKL with respect to SSPC frequency and functions and propose a new player in SSPC biology; future research will aim at the characterization of the signaling pathway downstream RANKL in this specific context.

In addition, to the best of our knowledge, our research provides for the first time the characterization of SSPC populations in two murine models of severe recessive osteopetrosis, where the drastic alteration of bone tissue microarchitecture and composition challenges the application of protocols established in WT animals. Taking advantage of our experience in the manipulation of murine models with extreme skeletal phenotypes, through this work we contribute to advance knowledge on RANKL-deficient osteopetrosis, which is a very rare subtype of the disease, even though it is not trivial translating this knowledge into benefit for these patients. In a different perspective, we envisage the significance of our results extends to the diverse pathological contexts with excessive bone loss where RANKL is a target of therapy as the main driver of bone resorption. The reduced mineralization capacity of human BMSCs treated in vitro with Denosumab during osteogenic induction raise some concerns about a putative effect on SSC functions in patients receiving this therapy, especially in the case of elderly individuals, which for physiological reasons already have lower SSC fitness. Indeed, also in the skeletal system, the natural process of aging is accompanied by a decline in the capacity of adult stem cells to maintain tissue integrity, owing to mechanisms only partially elucidated and comprising downregulation of Wnt signaling and of negative regulators of cellular senescence, skewed differentiation towards stroma, reduced transcriptomic diversity and epigenetic mechanisms [58]. Based on our results, the defective regenerative potential of SSCs in the elderlies might be further reduced by RANKL pharmacological blockade. According to this hypothesis, the reduction of osteoblast numbers recently reported in humanized RANKL mice treated with Denosumab [59] might be explained not only as consequence of the loss of crosstalk with osteoclasts, but also as a brake to SSC differentiation along the osteogenic lineage, in the presence of anti-RANKL treatment; in fact, the authors found also reduced formation of new osteocytes. Furthermore, the mechanism herein proposed borne by SSC might add to a direct effect on terminally differentiated cells and contribute to explain rebound bone loss and increased fractures upon Denosumab discontinuation. Indeed, Jähn-Rickert and colleagues recently showed that the density of empty osteocyte lacunae was higher in iliac crest bone biopsies of patients treated with Denosumab compared to treatment-naive individuals and remained high in trabecular bone after drug discontinuation, thus indicating that RANKL blockade led to sustained reduction of osteocyte viability [60].

## Conclusions

In conclusion, our work sheds light on a novel function of RANKL in the bone microenvironment and further confirms the importance of fine tuning the levels of this cytokine. Its role in the maintenance of SSC properties is relevant to the treatment of common bone pathologies such as osteoporosis and to the implementation of regenerative medicine approaches to skeletal disorders and deserves further investigation.

### Electronic supplementary material

Below is the link to the electronic supplementary material.

Supplementary Material 1

## Data Availability

All data needed to evaluate the conclusions of this work are present in the paper and/or in the supplemental information.

## Abbreviations

SSCs: Skeletal Stem Cells
RANKL: Receptor Activator of NF-kB Ligand
BMSCs: Bone marrow-derived Mesenchymal Stromal Cells
SSPCs: Skeletal Stem and Progenitor Cells
ocSSCs: Osteochondral Skeletal Stem Cells
pvSSCs: Perivascular Skeletal Stem Cells
preBCSPs: Pre-Bone, Cartilage and Stromal Progenitors
BCSPs: Bone, Cartilage and Stromal Progenitors
WT: Wild type
OPCs: Osteochondrogenic Progenitor Cells
APCs: Adipogenic Progenitor Cells
ARS: Alizarin Red Staining
OIM: Osteogenic Induction Medium
pOBs: Primary Osteoblasts
Dmab: Denosumab

## References

[1] Bianco P, Robey PG (2015). Skeletal stem cells. Development.

[2] Ortinau LC, Wang H, Lei K, Deveza L, Jeong Y, Hara Y, Grafe I, Rosenfeld SB, Lee D, Lee B (2019). Identification of functionally distinct Mx1 + αSMA + periosteal skeletal stem cells. Cell Stem Cell.

[3] Matsushita Y, Ono W, Ono N (2020). Skeletal stem cells for Bone Development and Repair: diversity matters. Curr Osteoporos Rep.

[4] Menon S, Salhotra A, Shailendra S, Tevlin R, Ransom RC, Januszyk M, Chan CKF, Behr B, Wan DC, Longaker MT (2021). Skeletal stem and progenitor cells maintain cranial suture patency and prevent craniosynostosis. Nat Commun.

[5] Xu J, Wang Y, Li Z, Tian Y, Li Z, Lu A, Hsu CY, Negri S, Tang C, Tower RJ (2022). PDGFRα reporter activity identifies periosteal progenitor cells critical for bone formation and fracture repair. Bone Res.

[6] Matsushita Y, Liu J, Chu AKY, Tsutsumi-Arai C, Nagata M, Arai Y, Ono W, Yamamoto K, Saunders TL, Welch JD (2023). Bone marrow endosteal stem cells dictate active osteogenesis and aggressive tumorigenesis. Nat Commun.

[7] Chan CK, Seo EY, Chen JY, Lo D, McArdle A, Sinha R, Tevlin R, Seita J, Vincent-Tompkins J, Wearda T (2015). Identification and specification of the mouse skeletal stem cell. Cell.

[8] Ambrosi TH, Sinha R, Steininger HM, Hoover MY, Murphy MP, Koepke LS, Wang Y, Lu WJ, Morri M, Neff NF (2021). Distinct skeletal stem cell types orchestrate long bone skeletogenesis. Elife.

[9] Pittenger MF, Discher DE, Péault BM, Phinney DG, Hare JM, Caplan AI (2019). Mesenchymal stem cell perspective: cell biology to clinical progress. NPJ Regen Med.

[10] Anderson DM, Maraskovsky E, Billingsley WL, Dougall WC, Tometsko ME, Roux ER, Teepe MC, DuBose RF, Cosman D, Galibert L (1997). A homologue of the TNF receptor and its ligand enhance T-cell growth and dendritic-cell function. Nature.

[11] Wong BR, Rho J, Arron J, Robinson E, Orlinick J, Chao M, Kalachikov S, Cayani E, Bartlett FS, Frankel WN (1997). TRANCE is a novel ligand of the tumor necrosis factor receptor family that activates c-Jun N-terminal kinase in T cells. J Biol Chem.

[12] Yasuda H, Shima N, Nakagawa N, Yamaguchi K, Kinosaki M, Mochizuki S, Tomoyasu A, Yano K, Goto M, Murakami A (1998). Osteoclast differentiation factor is a ligand for osteoprotegerin/osteoclastogenesis-inhibitory factor and is identical to TRANCE/RANKL. Proc Natl Acad Sci U S A.

[13] Kim N, Odgren PR, Kim DK, Marks SC, Choi Y (2000). Diverse roles of the tumor necrosis factor family member TRANCE in skeletal physiology revealed by TRANCE deficiency and partial rescue by a lymphocyte-expressed TRANCE transgene. Proc Natl Acad Sci U S A.

[14] Sobacchi C, Frattini A, Guerrini MM, Abinun M, Pangrazio A, Susani L, Bredius R, Mancini G, Cant A, Bishop N (2007). Osteoclast-poor human osteopetrosis due to mutations in the gene encoding RANKL. Nat Genet.

[15] Zhang N, Zhang ZK, Yu Y, Zhuo Z, Zhang G, Zhang BT (2020). Pros and Cons of Denosumab Treatment for Osteoporosis and implication for RANKL aptamer therapy. Front Cell Dev Biol.

[16] Tanaka S, Tanaka Y (2021). RANKL as a therapeutic target of rheumatoid arthritis. J Bone Min Metab.

[17] Huzum B, Antoniu S, Dragomir R (2022). Treatment of fibrous dysplasia: focus on denosumab. Expert Opin Biol Ther.

[18] Jayash SN, Hamoudi D, Stephen LA, Argaw A, Huesa C, Joseph S, Wong SC, Frenette J, Farquharson C (2023). Anti-RANKL therapy prevents glucocorticoid-Induced Bone loss and promotes muscle function in a mouse model of Duchenne muscular dystrophy. Calcif Tissue Int.

[19] Onji M, Penninger JM (2023). RANKL and RANK in Cancer Therapy. Physiol (Bethesda).

[20] Schena F, Menale C, Caci E, Diomede L, Palagano E, Recordati C, Sandri M, Tampieri A, Bortolomai I, Capo V (2017). Murine Rankl-/- mesenchymal stromal cells display an osteogenic differentiation defect improved by a RANKL-Expressing Lentiviral Vector. Stem Cells.

[21] Palagano E, Muggeo S, Crisafulli L, Tourkova IL, Strina D, Mantero S, Fontana E, Locatelli SL, Monari M, Morenghi E (2020). Generation of an immunodeficient mouse model of tcirg1-deficient autosomal recessive osteopetrosis. Bone Rep.

[22] Tondelli B, Blair HC, Guerrini M, Patrene KD, Cassani B, Vezzoni P, Lucchini F (2009). Fetal liver cells transplanted in utero rescue the osteopetrotic phenotype in the oc/oc mouse. Am J Pathol.

[23] Lo Iacono N, Blair HC, Poliani PL, Marrella V, Ficara F, Cassani B, Facchetti F, Fontana E, Guerrini MM, Traggiai E (2012). Osteopetrosis rescue upon RANKL administration to Rankl(-/-) mice: a new therapy for human RANKL-dependent ARO. J Bone Min Res.

[24] Lo Iacono N, Pangrazio A, Abinun M, Bredius R, Zecca M, Blair HC, Vezzoni P, Villa A, Sobacchi C (2013). RANKL cytokine: from pioneer of the osteoimmunology era to cure for a rare disease. Clin Dev Immunol.

[25] Sobacchi C, Abinun M (2022). Osteoclast-poor osteopetrosis. Bone.

[26] Palagano E, Menale C, Sobacchi C, Villa A (2018). Genetics of Osteopetrosis. Curr Osteoporos Rep.

[27] Capo V, Abinun M, Villa A (2022). Osteoclast rich osteopetrosis due to defects in the TCIRG1 gene. Bone.

[28] Li Q, Xu R, Lei K, Yuan Q (2022). Insights into skeletal stem cells. Bone Res.

[29] Guntur AR, Le PT, Farber CR, Rosen CJ (2014). Bioenergetics during calvarial osteoblast differentiation reflect strain differences in bone mass. Endocrinology.

[30] Schinke T, Schilling AF, Baranowsky A, Seitz S, Marshall RP, Linn T, Blaeker M, Huebner AK, Schulz A, Simon R (2009). Impaired gastric acidification negatively affects calcium homeostasis and bone mass. Nat Med.

[31] McKee C, Chaudhry GR (2017). Advances and challenges in stem cell culture. Colloids Surf B Biointerfaces.

[32] de Castro LF, Burke AB, Wang HD, Tsai J, Florenzano P, Pan KS, Bhattacharyya N, Boyce AM, Gafni RI, Molinolo AA (2019). Activation of RANK/RANKL/OPG pathway is involved in the pathophysiology of fibrous dysplasia and Associated with Disease Burden. J Bone Min Res.

[33] Zaheer S, LeBoff M, Lewiecki EM (2015). Denosumab for the treatment of osteoporosis. Expert Opin Drug Metab Toxicol.

[34] Ambrosi TH, Longaker MT, Chan CKF (2019). A revised perspective of skeletal stem Cell Biology. Front Cell Dev Biol.

[35] Ono N, Kronenberg HM (2015). Mesenchymal progenitor cells for the osteogenic lineage. Curr Mol Biol Rep.

[36] Pagani CA, Huber AK, Hwang C, Marini S, Padmanabhan K, Livingston N, Nunez J, Sun Y, Edwards N, Cheng YH (2021). Novel lineage-tracing system to identify site-specific ectopic bone precursor cells. Stem Cell Rep.

[37] Jeffery EC, Mann TLA, Pool JA, Zhao Z, Morrison SJ (2022). Bone marrow and periosteal skeletal stem/progenitor cells make distinct contributions to bone maintenance and repair. Cell Stem Cell.

[38] Baryawno N, Przybylski D, Kowalczyk MS, Kfoury Y, Severe N, Gustafsson K, Kokkaliaris KD, Mercier F, Tabaka M, Hofree M (2019). A Cellular Taxonomy of the bone marrow stroma in Homeostasis and Leukemia. Cell.

[39] Tikhonova AN, Dolgalev I, Hu H, Sivaraj KK, Hoxha E, Cuesta-Domínguez Á, Pinho S, Akhmetzyanova I, Gao J, Witkowski M (2019). The bone marrow microenvironment at single-cell resolution. Nature.

[40] Li B, Li J, Li B, Ouchi T, Li L, Li Y, Zhao Z (2023). A single-cell transcriptomic atlas characterizes age-related changes of murine cranial stem cell niches. Aging Cell.

[41] Zhang P, Dong J, Fan X, Yong J, Yang M, Liu Y, Zhang X, Lv L, Wen L, Qiao J (2023). Characterization of mesenchymal stem cells in human fetal bone marrow by single-cell transcriptomic and functional analysis. Signal Transduct Target Ther.

[42] Chan CKF, Gulati GS, Sinha R, Tompkins JV, Lopez M, Carter AC, Ransom RC, Reinisch A, Wearda T, Murphy M (2018). Identif Hum Skeletal Stem Cell Cell.

[43] Trompet D, Melis S, Chagin AS, Maes C. Skeletal stem and progenitor cells in bone development and repair. J Bone Min Res. 2024 Ahead of print. 10.1093/jbmr/zjae069 38696703

[44] Shen F, Huang X, He G, Shi Y (2023). The emerging studies on mesenchymal progenitors in the long bone. Cell Biosci.

[45] Bianco P, Minireview (2011). The Stem Cell Next Door: skeletal and hematopoietic stem cell niches. Bone Endocrinol.

[46] Kurenkova AD, Medvedeva EV, Newton PT, Chagin AS (2020). Niches for skeletal stem cells of mesenchymal origin. Front Cell Dev Biol.

[47] Sui X, Cai J, Li H, He C, Zhou C, Dong Y, Chen L, Zhang B, Wang Y, Zhang Y (2018). p53-dependent CD51 expression contributes to characteristics of cancer stem cells in prostate cancer. Cell Death Dis.

[48] Yao L, Li F, Yu C, Wang H, Wang Y, Ye L, Yu F (2023). Chronological and replicative aging of CD51+/PDGFR-α + pulp stromal cells. J Dent Res.

[49] Xie DM, Li YL, Li J, Li Q, Lu G, Zhai Y, Zhang J, Huang Z, Gao X (2019). CD51 distinguishes a subpopulation of bone marrow mesenchymal stem cells with distinct migratory potential: a novel cell-based strategy to treat acute myocardial infarction in mice. Stem Cell Res Ther.

[50] Cao Y, Kalajzic I, Matthews BG (2023). CD51 labels periosteal injury-responsive osteoprogenitors. Front Physiol.

[51] Lv FJ, Tuan RS, Cheung KM, Leung VY (2014). Concise review: the surface markers and identity of human mesenchymal stem cells. Stem Cells.

[52] Moraes DA, Sibov TT, Pavon LF, Alvim PQ, Bonadio RS, Da Silva JR, Pic-Taylor A, Toledo OA, Marti LC, Azevedo RB (2016). A reduction in CD90 (THY-1) expression results in increased differentiation of mesenchymal stromal cells. Stem Cell Res Ther.

[53] Xiong J, Onal M, Jilka RL, Weinstein RS, Manolagas SC, O’Brien CA (2011). Matrix-embedded cells control osteoclast formation. Nat Med.

[54] Logan M, Martin JF, Nagy A, Lobe C, Olson EN, Tabin CJ (2002). Expression of Cre Recombinase in the developing mouse limb bud driven by a prxl enhancer. Genesis.

[55] Ihida-Stansbury K, McKean DM, Gebb SA, Martin JF, Stevens T, Nemenoff R, Akeson A, Vaughn J, Jones PL (2004). Paired-related homeobox gene Prx1 is required for pulmonary vascular development. Circ Res.

[56] Ambrosi TH, Scialdone A, Graja A, Gohlke S, Jank AM, Bocian C, Woelk L, Fan H, Logan DW, Schürmann A (2017). Adipocyte Accumulation in the bone marrow during obesity and aging impairs stem cell-based hematopoietic and bone regeneration. Cell Stem Cell.

[57] Lee KW, Yeo SY, Gong JR, Koo OJ, Sohn I, Lee WY, Kim HC, Yun SH, Cho YB, Choi MA (2022). PRRX1 is a master transcription factor of stromal fibroblasts for myofibroblastic lineage progression. Nat Commun.

[58] Butler MGK, Ambrosi TH, Murphy MP, Chan CKF (2022). Aging of skeletal stem cells. Adv Geriatr Med Res.

[59] Fu Q, Bustamante-Gomez NC, Reyes-Pardo H, Gubrij I, Escalona-Vargas D, Thostenson JD, Palmieri M, Goellner JJ, Nookaew I, Barnes CL (2023). Reduced osteoprotegerin expression by osteocytes may contribute to rebound resorption after denosumab discontinuation. JCI Insight.

[60] Jähn-Rickert K, Wölfel EM, Jobke B, Riedel C, Hellmich M, Werner M, McDonald MM, Busse B (2020). Elevated bone hardness under Denosumab Treatment, with persisting lower osteocyte viability during discontinuation. Front Endocrinol (Lausanne).

