# Supplementary material for: Rankl genetic deficiency and functional blockade undermine skeletal stem and progenitor cell differentiation
Source: Stem Cell Res Ther. 2024 Jul 6;15:203. doi: 10.1186/s13287-024-03803-3 (PMC11227705; doi:10.1186/s13287-024-03803-3)
Supplement: Supplementary file 1 — Supplementary Material 1 [file 13287_2024_3803_MOESM1_ESM.docx]

**Schiavone et al., Supplementary Information**

**Supplementary Methods**

**Western blot analysis**

After 7 and 14 days of culture in the presence or absence of osteogenic induction medium, primary osteoblasts were lysed in Cell Lysis Buffer (Cell Signaling). Protein concentrations were estimated using the BCA Pierce Assay Kit (Thermo Fisher following the manufacturer’s instructions, on a Synergy H4 instrument (BioTek Instruments, Inc.). Twenty micrograms of each protein extract were separated on a 10% Sodium Dodecyl Sulfate Polyacrylamide Gel Electrophoresis (SDS-PAGE), transferred to a nitrocellulose membrane, and probed with a monoclonal antibody specific for mouse Lgr4 (Santa Cruz), washed and probed with a secondary antibody conjugated with HRP and developed using the Thermo Scientific Super Signal ECL (Thermo Fisher). Then, the same membrane was incubated with a monoclonal antibody specific for mouse Rank (Abcam), washed, probed with the HRP-conjugated secondary antibody, and developed as above. Images were captured using the ChemiDoc MP Imaging System equipped with Image Lab Software (Bio-Rad).

**Legends to Supplementary Figures**

**Fig. S1** Characterization of ocSSPCs isolated from the skull of 5-week-old WT and *Rankl^-/-^* mice. Expression level of the positive markers in the indicated cell populations, expressed as mean fluorescence intensity (MFI). Data are expressed as mean ± SEM. * p < 0.05.


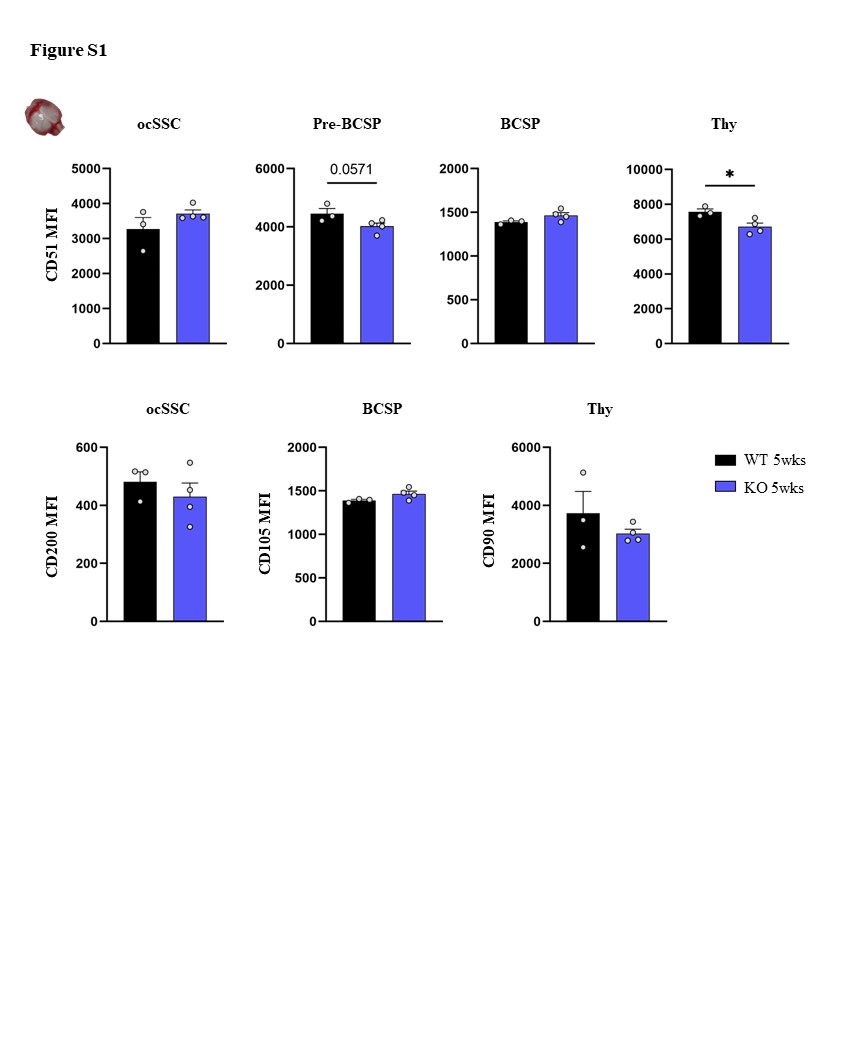


**Fig. S2** Characterization of the osteogenic potential of WT and *Rankl^-/-^* (KO) ocSSCs and primary osteoblasts. Higher magnification of the representative image of ARS-stained cultures shown in Fig. 4 in the Main text.


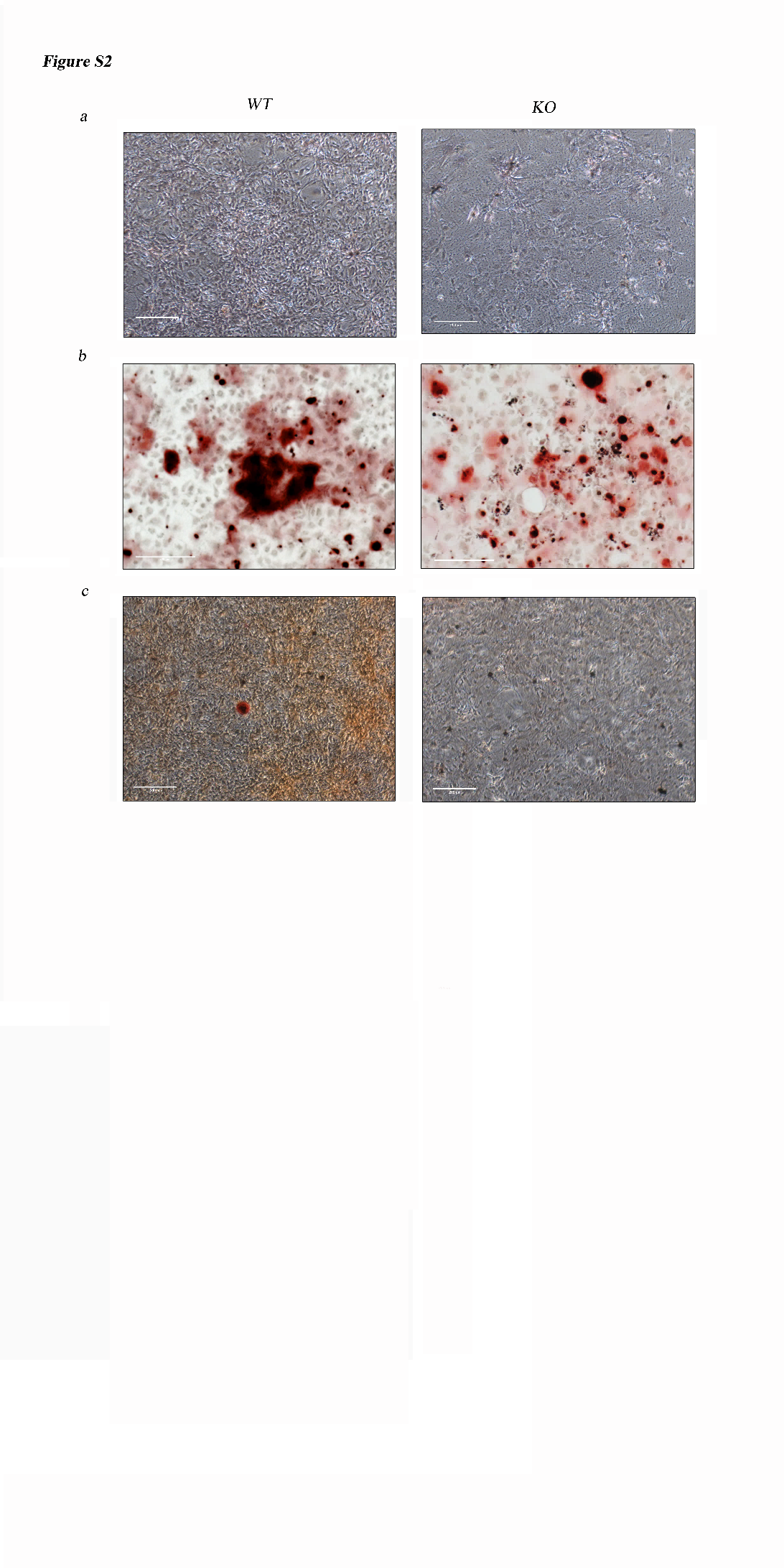


**Fig. S3** Characterization of the osteogenic potential of WT and *Rankl^-/-^* (KO) primary osteoblasts after 7 days of osteogenic induction. **a** WT and KO primary osteoblast cultures were established and induced to mineralize according to standard protocols. Representative images after ARS and quantization of the extracted stain by Abs reading at 405 nm. Scale bars: 500 and 200 μm (left and right panels, respectively). **b** Gene expression analysis of osteogenic marker genes in WT and KO osteoblasts, normalized on *Gapdh* and expressed as Arbitrary Units (A.U.). All the data are expressed as mean ± SEM. * p < 0.05. **c** Western blot analysis of Lgr4 and Rank (and Actin as housekeeping) in protein extracts from WT and KO primary osteoblasts after 7 and 14 days of osteogenic induction or in the absence of osteogenic induction medium (OIM).


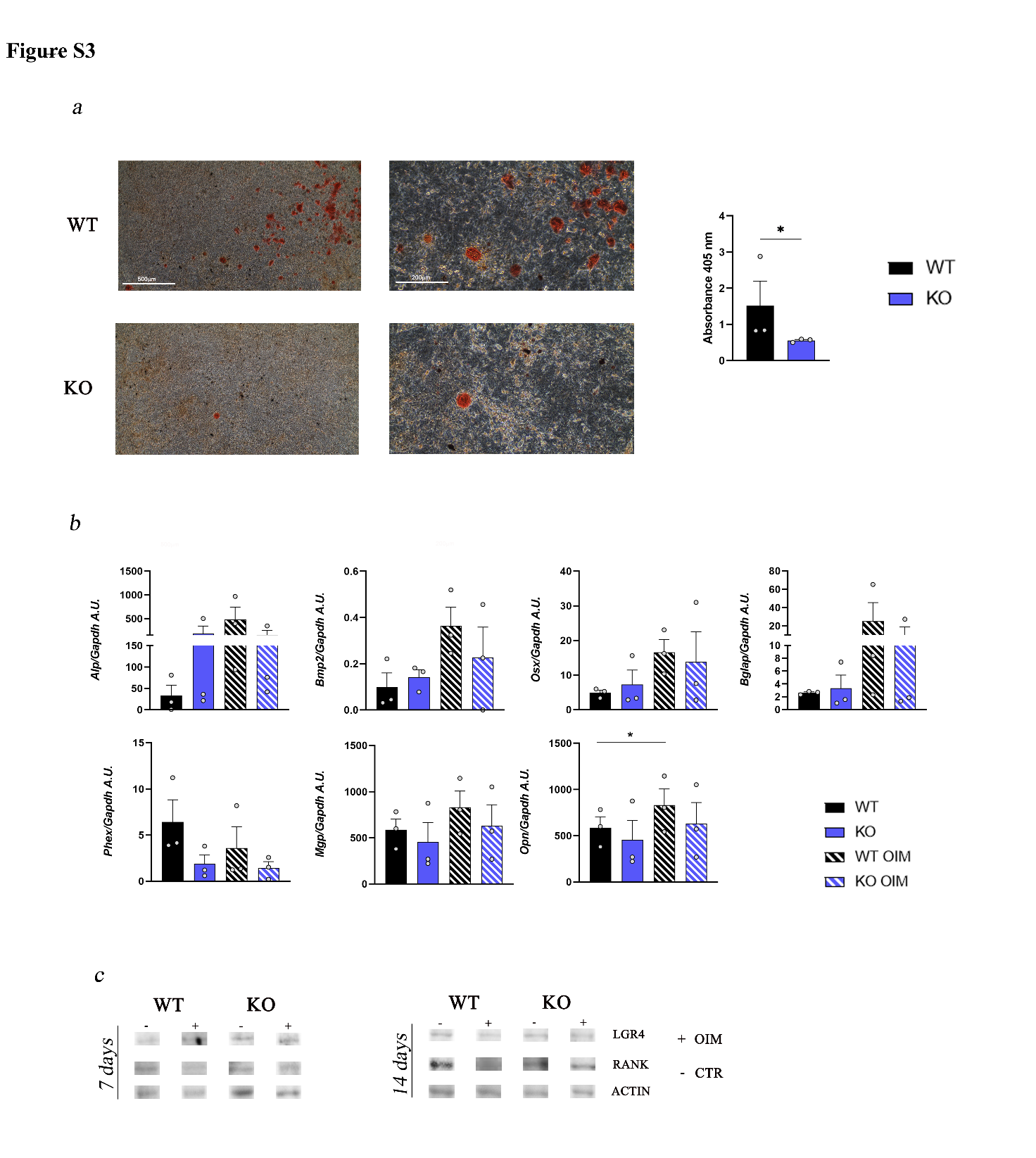


**Fig. S4 a** Assessment of the impact of Denosumab treatment on the cell viability of human BMSCs from healthy donors. Cell viability was evaluated by MTT assay at different timepoints. Quantization was done by absorbance (Abs) reading at 560 nm. Data are expressed as mean ± SEM. OIM: Osteogenic Induction Medium. ISO: isotype control. Dmab: Denosumab. **b** Impact of Denosumab treatment on the mineralization capacity of human BMSCs. Higher magnification of the representative image of ARS-stained cultures of human BMSCs from healthy donors after 2 weeks of culture either in basal medium (CTR) or in OIM alone or in OIM + isotype control (OIM + ISO) or in OIM + Denosumab (OIM +Dmab), shown in Fig. 6 in the Main text.


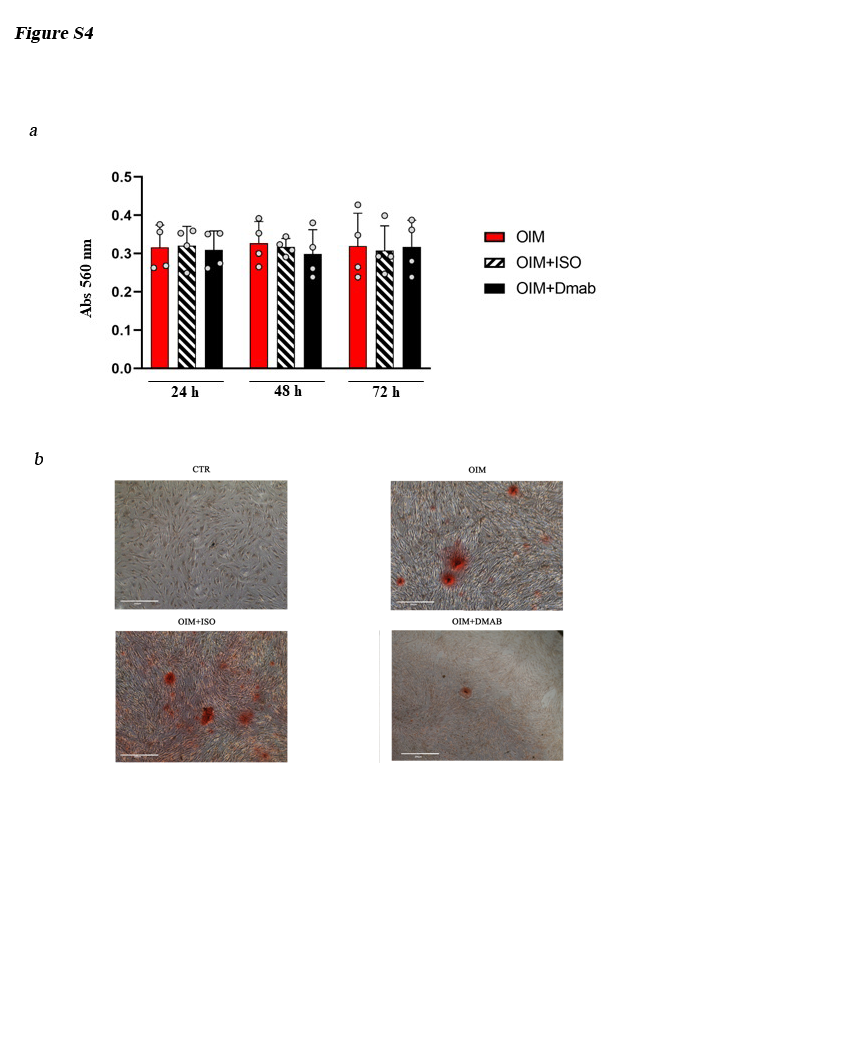


**Table S1** Fluorescent-conjugated antibodies used for fractionation by fluorescence activated-cell sorting of ocSSCs.

| **Antibody** | **Fluorophore** | **Code** | **Company** | **Dilution** |
| --- | --- | --- | --- | --- |
| CD51 | Biotin | 551380 | BD | 1:50 |
| CD45 | PE-Cy7 | 25-0451-82 | eBioscience | 1:1000 |
| Ter119 | PE-Cy7 | 25-5921-82 | eBioscience | 1:400 |
| Tie2 | APC | 124010 | Biolegend | 1:50 |
| Thy1.2/CD90.2 | APC-Fire 750 | 105347 | Biolegend | 1:200 |
| CD200 | PerCP | 46-5200-82 | eBioscience | 1:200 |
| CD105 | Pacific Blue | 57-1051-82 | eBioscience | 1:100 |
| 6C3/BP-1 | PE | 12-5891-81 | eBioscience | 1:50 |
| SA | FITC | 554460 | BD | 1:400 |

**Table S2** Fluorescent-conjugated antibodies used for fractionation by fluorescence activated-cell sorting of pvSSCs.

| **Antibody** | **Fluorophore** | **Code** | **Company** | **Dilution** |
| --- | --- | --- | --- | --- |
| CD31 | PE | 12-0311-83 | eBioscience | 1:100 |
| CD45 | PE | 12-0451-82 | eBioscience | 1:1000 |
| CD140a/PDGFRα | APC | 17-1401-81 | eBioscience | 1:100 |
| CD24 | eFluor 450 | 48-0242-80 | eBioscience | 1:100 |
| Sca1 | FITC | 11-5981-82 | eBioscience | 1:100 |

**Table S3** Gene-specific primers used in gene expression analysis of murine cells

| **Gene** | **Primer sequence** |
| --- | --- |
| *Gapdh* | F: 5’-AGGTCGGTGAACGGATTTG-3’  R: 5’-TGTAGACCATGTAGTTGAGGTCA-3’ |
| *Oct4* | F: 5’-GATGCGGACTGTGTTCTCTC-3’  R: 5’-GCTTGCACTTCATCCTTTGG-3’ |
| *Nanog* | F: 5’-CACAGTTTGCCTAGTTCTGAGG-3’  R: 5’-GCAAGAATAGTTCTCGGGATGAA-3’ |
| *Sox2* | F: 5’-CACATG AAG GAGCACCCGGATTAT-3’  R: 5’-GTTCATGTGCGCGTAACTGTCCAT-3’ |
| *Rank* | F: 5’-GAAGGCTCATGGTTGGATGT-3’  R: 5’-GTAGCCCAAGGGTATTTCAG-3’ |
| *Lgr4* | F: 5’-AAGATAACAGCCCCCAAGAC-3’  R: 5’-AGGCAGTGATGAACAAGACG-3’ |
| *Alp* | F: 5’-AAGGCTTCTTCTTGCTGGTG-3’  F: 5’-GGTGTATCCACCGAATGTGA-3’ |
| *Bmp2* | F: 5’-CGGACTGCGGTCTCCTAA-3’  R: 5’-GGGGAAGCAGCAACACTAGA-3’ |
| *Osx* | F: 5’-CTCTCCTGCAGGCAGTCCT-3’  R: 5’GGGAAGGTGGGTAGTCAT3’ |
| *Bglap* | F: 5’-AAGCAGGAGGGCAATAA-3’  R: 5’-TTTGTAGGCGGTCTTCAAGC-3’ |
| *Phex* | F: 5’-ATGGCTGGATAAGCAATAACCC-3’  R: 5’-TCAACTTGAGGTCAACATTGTGT-3’ |
| *Mgp* | F: 5’-AGCCCAAAAGAGAGTCCAGGA-3’  R: 5’-TGCCTGAAGTAGCGGTTGTAG-3’ |
| *Opn* | F: 5’-TTTACAGCCTGCACCCAGAT-3’  R: 5’-CAGATTCATCCGAGTCCACA-3’ |

**Table S4** Gene-specific primers used in gene expression analysis of human cells

| **Gene** | **Primer sequence** |
| --- | --- |
| *18S* | F: 5’-cgcagctaggaataatggatagg-3’  R: 5’-catggcctcagttccgaaa-3’ |
| *RUNX2* | F: 5’-cagtgacaccatgtcagcaa-3’  R: 5’-gctcacgtcgcctcattttg-3’ |
| *SPP1* | F: 5’-gagggcttggttgtcagc-3’  R: 5’-caattctcatggtagtgagttttcc-3’ |
